# A mean field model for movement induced changes in the beta rhythm

**DOI:** 10.1007/s10827-017-0655-7

**Published:** 2017-07-26

**Authors:** Áine Byrne, Matthew J Brookes, Stephen Coombes

**Affiliations:** 1 Centre for Mathematical Medicine and Biology, School of Mathematical Sciences, University of Nottingham, University Park, Nottingham, NG7 2RD UK; 2 Sir Peter Mansfield Imaging Centre, School of Physics and Astronomy, University of Nottingham, University Park, Nottingham, NG7 2RD UK

**Keywords:** Post-movement beta rebound, Movement related beta decrease, Neural mass, Synchrony, Power spectra, Magnetoencephalography, MEG, Electroencephalography, EEG, Mean field

## Abstract

In electrophysiological recordings of the brain, the transition from high amplitude to low amplitude signals are most likely caused by a change in the synchrony of underlying neuronal population firing patterns. Classic examples of such modulations are the strong stimulus-related oscillatory phenomena known as the movement related beta decrease (MRBD) and post-movement beta rebound (PMBR). A sharp decrease in neural oscillatory power is observed during movement (MRBD) followed by an increase above baseline on movement cessation (PMBR). MRBD and PMBR represent important neuroscientific phenomena which have been shown to have clinical relevance. Here, we present a parsimonious model for the dynamics of synchrony within a synaptically coupled spiking network that is able to replicate a human MEG power spectrogram showing the evolution from MRBD to PMBR. Importantly, the high-dimensional spiking model has an exact mean field description in terms of four ordinary differential equations that allows considerable insight to be obtained into the cause of the experimentally observed time-lag from movement termination to the onset of PMBR (∼ 0.5 s), as well as the subsequent long duration of PMBR (∼ 1 − 10 s). Our model represents the first to predict these commonly observed and robust phenomena and represents a key step in their understanding, in health and disease.

## Introduction

The modelling of brain rhythms is now a well established and vibrant part of computational neuroscience. Ever since the first recordings of the human electroencephalogram (EEG) in 1924 by Berger (1929) electrophysiological brain recordings have been shown to be dominated by oscillations (rhythmic activity in cell assemblies) across a wide range of temporal scales and scientists have sought to develop large scale models to describe the five main frequency bands of delta (1 − 4 Hz), theta (4 − 8 Hz), alpha (8 − 13 Hz), beta (13 − 30 Hz) and gamma (30 − 200 Hz). Moreover, it has long been known, since the early works of Jasper and Andrews (1936, 1938), that different brain rhythms can be localised to specific areas of the brain, and that these rhythms can be functionally distinct. For example, they showed that the beta rhythm present in the vicinity of the central sulcus was not affected by the presentation of a weak visual stimulus which suppressed the alpha rhythm, recorded from the occipital lobe. Given the challenge of modelling such complex behaviour it is perhaps no surprise that the long and industrious history of brain modelling has delivered more than one tool for this job. For issues that relate to spike times and their synchrony we can appeal to conductance based modelling, large scale network simulations and theories for understanding coupled oscillators, as recently surveyed in Ashwin et al. (2016). For questions that relate to understanding the coarse grained activity of either synaptic currents, mean membrane potentials or population firing rates, it is more natural to appeal to neural mass models, as reviewed in Coombes (2010). Indeed the latter have proven especially fruitful in providing large scale descriptions of how neural activity evolves over both space as well as time (Coombes et al. 2014; Pinotsis et al. 2014). However, these two approaches are dangerously close to creating a dichotomy so that there is no ideal computational modelling framework for understanding the role of spike-timing in generating localised brain rhythms.

A case in point that challenges the modelling tools currently available to us is the work of Jasper and Penfield (1949) who showed that beta rhythms generated from the motor cortex are suppressed during voluntary movement. This phenomenon is known as movement related beta decrease (MRBD). It wasn’t until some years later that the post-movement beta rebound (PMBR) (a temporary rise in amplitude of beta oscillations following movement cessation) was discovered (Riehle and Vaadia 2004; Pfurtscheller et al. 1996; Jurkiewicz et al. 2006). MRBD usually lasts for approximately 0.5 seconds and PMBR can last for up to several seconds. MRBD and PMBR are extremely robust, with clear amplitude changes in individual subjects and trials (Pfurtscheller and Lopes da Silva 1999). Interestingly, similar effects have been seen in studies where the subject is asked to think about moving, without carrying out the movement (Schnitzler et al. 1997; Pfurtscheller et al. 2005). These beta band modulations are believed to be caused by changes of synchrony within a relatively localised region of motor cortex (Stancák and Pfurtscheller 1995). Hence, MRBD is regarded as a special case of event-related desynchronisation (ERD) and PMBR a special case of event-related synchronisation (ERS).

Multiple papers have employed a large number of carefully controlled paradigms, in humans and animals to further investigate beta rebound phenomena and their modulations by tasks (see Cheyne 2013; Kilavik et al. 2013 for reviews). However, despite the robust nature of the beta task induced decrease and post stimulus rebound, the effect itself is relatively poorly understood and, at the time of writing, there has been, to our knowledge, no computational model capable of describing the beta rebound. In general, high amplitude beta oscillations are thought to reflect inhibition (Cassim et al. 2001; Gaetz et al. 2011), a hypothesis supported by quantifiable relationships between beta amplitude and local concentrations of the inhibitory neurotransmitter gamma aminobutyric acid (GABA) (Gaetz et al. 2011; Hall et al. 2011; Jensen et al. 2005; Muthukumaraswamy et al. 2013). This means that the observed MRBD might reflect an increase in processing during movement planning and execution and the PMBR might reflect active inhibition of neuronal networks post movement (Alegre et al. 2008; Solis-Escalante et al. 2012). An alternative, but not mutually exclusive hypothesis which has been proposed by Donner and Siegel (2011) (also outlined in Liddle et al. 2016) is that the beta signal, in part, represents long range integration across multiple brain regions (see also Liddle et al. 2013). Indeed this is a hypothesis supported by some evidence suggesting that large scale distributed network connectivity is mediated by beta oscillations (Brookes et al. 2011; Hall et al. 2014; Hipp et al. 2012).

To describe beta rebound we are faced with modelling a mesoscopic brain scale and in particular the changes of synchrony within a population of say 10^6−7^ excitatory pyramidal cells and their associated inhibitory interneurons. A neural mass model would be ideal for this scale, if the question of interest related to rate rather than spike, which suggests instead a simulation of a spiking neural network model. Unfortunately the latter can be notoriously hard to gain insight from for very large numbers of neurons. Ideally we would have access to a statistical neurodynamics providing a bridge between the two levels of description. This is an open mathematical problem. However, recent progress in obtaining a mean field reduction for a very specific choice of large scale spiking model has been made, and is ideally suited as a basis for breaking the dichotomy noted above. The single neuron model of choice being either a *θ*-neuron (So et al. 2014; Luke et al. 2013) or a (formally equivalent) quadratic integrate-and-fire (QIF) neuron (Pazó and Montbrió 1009), and the coupling being global and mediated by pulses (namely instantaneous synapses). Given the dense connections of connections in cortex on small scales (Klinshov et al. 2014) the global coupling assumption is not so restrictive for our purposes, though the assumption of fast synapses should be relaxed to incorporate more realistic post synaptic responses. This is precisely the issue we address here to develop a model capable of explaining MRBD and PMBR.

In what follows, Section 2 gives a recapitulation of cortical rebound, illustrated with newly acquired magnetoencephalography (MEG) data, along with some recently published results. A candidate large scale computational model is described in Section 3, utilising realistic synaptic conductance changes. The model is cast in both voltage and phase variable so that it can be understood both as a QIF network, and also as a phase-oscillator network so that a connection to Kuramoto type networks can be made. Importantly we develop an exact low dimensional mean field description capable of capturing the behaviour of a globally coupled network in the limit of a large number of neurons. The macroscopic variables of interest are now firing rate and mean membrane potential (for the voltage description) or the Kuramoto measure of synchrony (for the phase description). Importantly we show in Section 4 that the response of the mean field model to stimulation leads to spectrograms with all of the key features observed in MRBD and PMBR. Finally in Section 5 we emphasise the benefits of this new type of neural mass model, capable of tracking not only changes in firing rate but also coherence within a population, in describing cortical rebound, as well as discuss natural extensions to our initial single population approach.

## MRBD and PMBR: a recapitulation

**Fig. 1:**
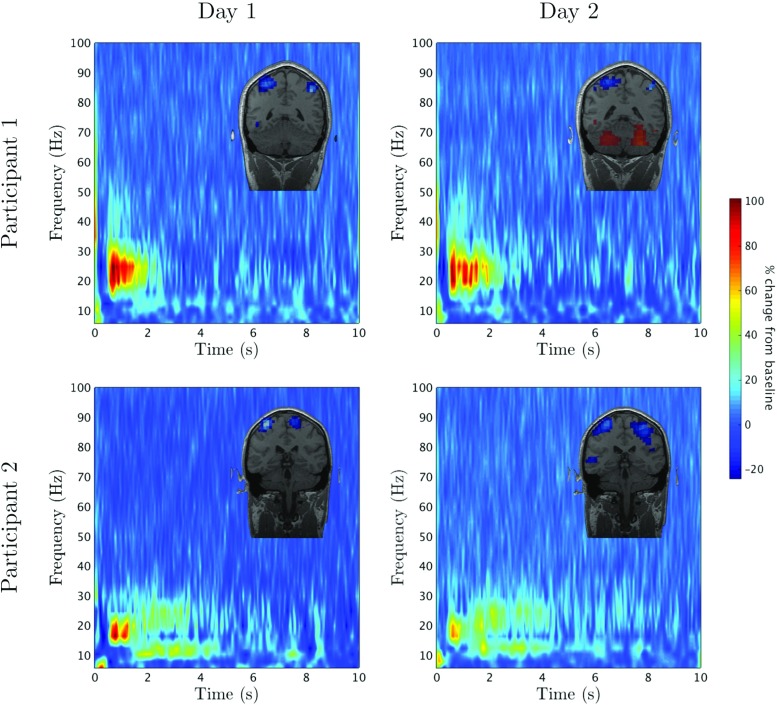
Robust beta rebound for median nerve stimulation: Time-frequency spectrograms showing the percentage change from baseline of the activity in the motor cortex, for 2 participants on two separate days. The *top row* shows the results for participant 1 and the *bottom row* represents data from participant 2. Each participant displayed a clear difference in their PMBR. However, the strength and timing of both the MRBD and PMBR are consistent between subjects and trials. PMBR for each participant appears to be reproducible

Beta band modulation is robust across subjects; occurring during internally and externally cued movements, as well as during somatosensory stimulation. To demonstrate this, for somatosensory stimulation, we carried out a series of median nerve stimulation experiments on two healthy participants. The participant’s median nerve was stimulated at the wrist using a constant current stimulator and the neurological response was measured using MEG (for more details on the experimental design see Appendix A). The experiment was repeated on separate days to determine how reproducible the results were. Figure 1 shows the relative time-frequency spectrograms for two such experiments for each participant, where baseline activity has been subtracted. The top line represents the data for participant 1 and the bottom line represents participant 2. For each case there is a 10–20% decrease in power for ∼ 0.5 s, demonstrating MRBD. At ∼ 0.5 s there is a 60–100% increase in power, exemplifying PMBR. Although the comparison between participants shows dissimilarities in the shape and length of PMBR, the strength and timing of both the MRBD and PMBR are comparable. Importantly, the similarity between each participant’s time-frequency spectrogram on separate days is compelling.

**Fig. 2:**
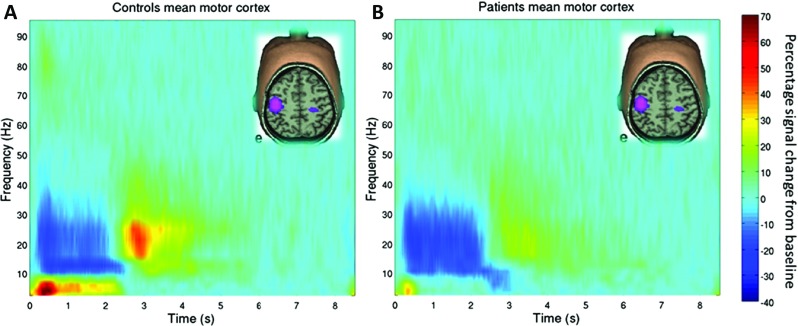
The beta rebound and its disruption in patients with schizophrenia: (**a**) Time-frequency spectrograms showing changes in the amplitude of neural oscillations, in contralateral sensorimotor cortex, when subjects execute a 2s finger movement. Note that, in the beta band the loss in oscillatory power during movement is accompanied by an increase in power on movement cessation. (**b**) Equivalent time-frequency spectrogram in patients with schizophrenia. Note the significant reduction in PMBR (Figure reproduced with permission from Robson et al. 2015.)

**Fig. 3:**
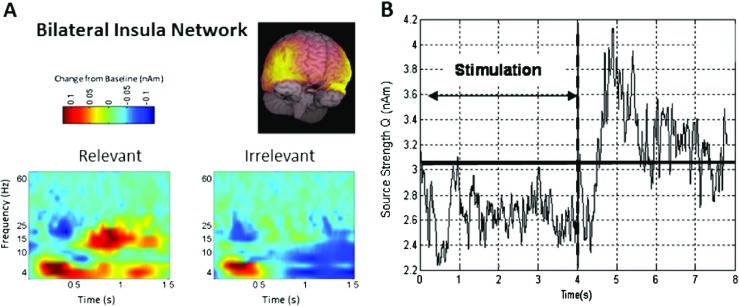
Task induced beta band decrease and rebound phenomena in other cortical regions: (**a**) Time-frequency dynamics in a network of brain areas including bilateral insula. The task involved visual stimuli that were relevant and irrelevant to the task. Note the significant reduction and rebound in beta oscillations in the relevant condition. (Reproduced with permission from Liddle et al. 2016.) (**b**) Timecourse showing the envelope of beta oscillations in primary visual cortex during passive viewing of a visual grating. Visual stimulation occurred in the 0 s to 4 s window. Note again the task induced power loss and post stimulus rebound (Reproduced with the authors’ permission from Stevenson et al. 2011.)

Recent work, reviewed in Brookes et al. (2011, 2012) and Robson et al. (2015), has begun to show the potential importance of beta band modulation. For example, Fig. 2 (recreated with permission from Robson et al. 2015) shows relative time-frequency spectra depicting the changes in neural oscillations in sensorimotor cortex in response to a cued finger movement task. The left hand panel (a) shows the case for healthy individuals. The time-frequency spectrum is extracted from a location of interest in left primary motor cortex. Notice that in the beta band, the MRBD and PMBR are observed clearly. The right hand panel (b) shows the case for patients with schizophrenia. Note the significant reduction in PMBR. Furthermore, this same study showed that the magnitude of the beta rebound correlated significantly with the severity of ongoing symptoms of schizophrenia, thus highlighting direct clinical relevance to the measurement. This is just one example of how beta band oscillations have been identified as a potential biomarker of disease; other examples include Parkinson’s Disease (Timmermann and Florin 2012). In addition, the robustness of MRBD and PMBR has meant that they have also been used in neuroscience applications ranging from brain computer interfaces (Pfurtscheller and Solis-Escalante 2009) to markers of neural plasticity (Gaetz et al. 2010; Mary et al. 2015). It is also noteworthy that the beta band power loss and rebound, whilst commonly thought of as being observable in the sensorimotor cortex, is not a sole property of the sensorimotor system. For example, Fig. 3 shows instances of observation of very similar phenomena in other cortical areas. Figure 3a shows the time-frequency oscillatory dynamics of a network of brain areas encapsulating bilateral insula cortex, throughout a cognitive task (Liddle et al. 2016; Brookes et al. 2012). The task itself involves presentation of a series of visual stimuli; some stimuli are relevant to the task, others irrelevant. Subjects were asked to respond if the relevant stimuli match some predetermined condition. Note here that only the non-target stimuli are shown (meaning that the subjects did not actually make a response). In the relevant condition, clear beta modulation is observed with a decrease in amplitude followed by a rebound above baseline. Furthermore, this effect was also shown to be abnormal in schizophrenia, again demonstrating its clinical relevance. Figure 3b shows the case for simple sensory stimulation of the visual cortex (Stevenson et al. 2011). Here, subjects were asked to passively view a drifting visual grating; the figure shows the envelope of beta band oscillations throughout the task. Note again the clear structure with a loss in beta amplitude during stimulation and an increase on stimulus cessation. These represent two simple examples which show that the beta band effect is not simply a property of the sensorimotor system, but rather is a ubiquitous effect that is observed robustly across many cortical regions.

The above indicates that stimulus related beta power loss and post stimulus rebound are generally observable effects, seen in many cortical areas, during both sensory and cognitive tasks. Further, the reduction of rebound in disease has been robustly demonstrated. Thus, the generation of new mathematical models from which we can accurately predict task induced beta band dynamics are of interest.

## A mean field model for spiking networks

There are a now a plethora of single neuron models for describing the spiking dynamics of cortical cells, many of which are extensions of the basic Hodgkin-Huxley model to incorporate nonlinear ionic currents that allow low frequency firing in response to constant current injection. Importantly mathematical neuroscience has identified a number of mechanisms that can generate ‘f-I’ curves with this property, with perhaps the most well known being the saddle-node on an invariant circle (SNIC) bifurcation (Ermentrout and Kopell 1986). This has led to the formulation of the elegant *θ*-neuron model (or Ermentrout-Kopell canonical model) which can mimic the firing and response properties of a cortical cell with a purely one dimensional dynamical system evolving on a circle. In a certain limit this is formally equivalent to the quadratic integrate-and-fire (QIF) model, also designed explicitly for understanding the generation of low firing rates in cortex (Latham et al. 2000). Given the simplicity of these models they are a natural candidate for cortical network studies, not only because they are computationally cheap, but because there is more chance to develop a statistical neurodynamics for such models than their biophysically complicated conductance based counterparts. Indeed mean field models for globally pulse-coupled networks have recently been developed by Luke et al. (2013) for *θ*-neuron models, and by Montbrió et al. (2015) for QIF models. To make these models more relevant to the interpretation of brain imaging signals, and in particular EEG and MEG, it is vital to augment the networks with more realistic forms of synaptic interaction and to move away from the overly restrictive assumption of fast pulsatile synaptic currents.

We consider a network of *N* QIF neurons each with a voltage *v*_*i*_, for *i* = 1, … , *N*, evolving according according to the following set of coupled ordinary differential equations (ODEs):

1 $$ C \frac{\mathrm{d} }{\mathrm{d} t} v_{i} = \eta_{i} + \kappa {v_{i}^{2}} + I_{i}, \qquad i=1,\ldots, N . $$

Here *C* is a capacitance, *η*_*i*_ is a constant current drive, *κ* is a proportionality constant, which from now on (without loss of generality) will be set to unity, and *I*_*i*_ is the synaptic current,

2 $$ I_{i} = g(t) (v_{\text{syn}}-v_{i}), $$

where *g*(*t*) represents a common time-dependent synaptic drive, which we shall take to arise through global coupling. This acts to push the voltage toward the synaptic reversal potential *v*_syn_. If the synaptic current is positive (negative) we say that the synapse is excitatory (inhibitory). The QIF network has discontinuous trajectories since whenever *v*_*i*_ reaches a threshold value *v*_th_ it is *reset* to the value *v*_reset_. This firing condition is also used to define an implicit set of firing times according to $v_{i}({T_{i}^{m}})=v_{\text {th}}$, where ${T_{i}^{m}}$ denotes the *m* th firing time of the *i* th neuron. These in turn can be used to generate a set of conductance changes for the *i* th neuron that we write in the form ${\sum }_{m \in \mathbb {Z}} s (t-{T_{i}^{m}})$, where *s*(*t*) is a fixed temporal filter. For a globally coupled network, with strength of synapse *k*/*N*, the total synaptic conductance change at each neuron is then

3 $$ g(t) = \frac{k}{N} \sum\limits_{j=1}^{N} \sum\limits_{m \in \mathbb{Z}} s(t-{T_{j}^{m}}) . $$

For fast pulsatile interactions we may set *s*(*t*) = *δ*(*t*), where *δ* is a Dirac-delta function. For a more realistic form describing a normalised post synaptic potential (PSP) with an exponential decay we may set *s*(*t*) = *α* e^−*αt*^Θ(*t*), whilst for a more general PSP with both a rise and fall time we would set $s(t)=(1/\alpha _{1} - 1/\alpha _{2})^{-1} [\alpha _{1} \mathrm {e}^{-\alpha _{1} t} - \alpha _{2} \mathrm {e}^{-\alpha _{2} t}]{\Theta }(t)$. Here Θ(*t*) is a Heaviside step function included to enforce causality, and the parameters *α*, *α*_1,2_ are decay rates. Exploiting the fact that in all these cases *s*(*t*) is the Green’s function of a linear differential operator *Q* then we may also write *g* as the solution to the ODE system

4 $$ Q g(t) = \frac{k}{N} \sum\limits_{j=1}^{N} \sum\limits_{m \in \mathbb{Z}} \delta (t-{T_{j}^{m}}) . $$

For the corresponding operator *Q* to the choice of *s* see Table 1. For the rest of this paper we shall work with the choice *s*(*t*) = *α* ^2^ *t* e^−*αt*^Θ, describing the so-called *α*-function. This can be obtained from the difference of exponentials form described above in the limit *α*_1,2_ → *α*, so that the corresponding differential operator *Q* is

5 $$ Q=\left( 1 + \frac{1}{\alpha} \frac{\mathrm{d} }{\mathrm{d} t} \right)^{2} . $$

**Fig. 4:**
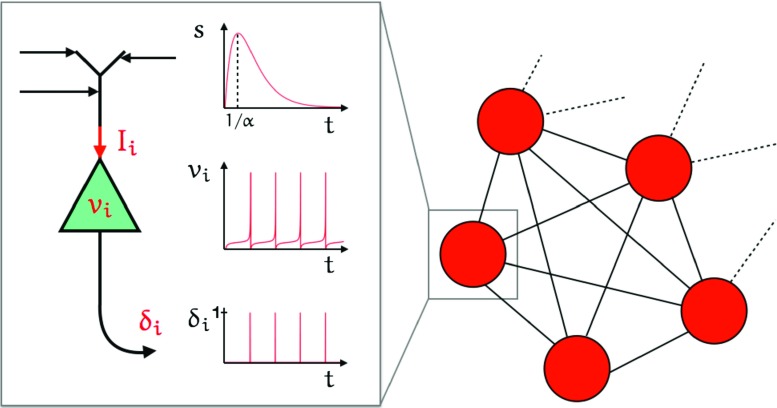
Neural network: The diagram on the right shows an all-to-all coupled network. The *zoom on the left* shows each of the components of Eqs. (1), (3) and (4). The *top plot of the zoom* shows the shape of the synaptic filter for the case that *s*(*t*) is an *α*-function: *s*(*t*) = *α* ^2^ *t* e^−*αt*^Θ, where 1/*α* is the *time-to-peak*. *I*_*i*_ is the total synaptic current that enters the cell body and *v*_*i*_ is the voltage of the cell which oscillates as shown in the *middle plot*. The corresponding output spike train is given by a sequence of Dirac-delta functions $\delta _{i} = {\sum }_{m \in \mathbb {Z}} \delta (t-{T_{i}^{m}})$, as illustrated in the *bottom plot*

Thus Eqs. (1)–(5) define our spiking model. This is further illustrated in Fig. 4 for an all-to-all coupled neural network, with an inset showing the single neuron dynamics. Each neuron generates a train of spikes described by a sequence of Dirac-delta functions that are then filtered with the kernel *s*(*t*) to generate a synaptic current according to Eq. (2). From this one may in principle use Maxwell’s equations to determine the magnetic field that would underly an MEG-like signal. However, for simplicity we shall take the average network current to be a proxy for this physiological signal. This is given explicitly by *g*(*t*)(*v*_syn_ − *V* (*t*)), where

6 $$ V(t) = \frac{1}{N} \sum\limits_{j=1}^{N} v_{j}(t) , $$

which describes the average membrane potential.

**Table 1:** Synaptic filtering: Examples of differential operators and their corresponding temporal filters

*Q*	*s*(*t*)
1	*δ*(*t*)
$\displaystyle \left (1 \,+\, \frac {1}{\alpha } \frac {\mathrm {d} }{\mathrm {d} t} \right )$	$\displaystyle \alpha \mathrm {e}^{-\alpha t} {\Theta }(t)$
$\displaystyle \left (1 \,+\, \frac {1}{\alpha _{1}} \frac {\mathrm {d} }{\mathrm {d} t} \right ) \left (1 \,+\, \frac {1}{\alpha _{2}} \frac {\mathrm {d} }{\mathrm {d} t} \right )$	$\displaystyle \left (\frac {1}{\alpha _{1}} \,-\, \frac {1}{\alpha _{2}} \right )^{-1} \left [\alpha _{1} \mathrm {e}^{-\alpha _{1} t} \,-\, \alpha _{2} \mathrm {e}^{-\alpha _{2} t} \right ]{\Theta }(t)$

The first example shows pulsatile coupling, where no synaptic filtering has been applied to the incoming spike. The second type of filter shown is an exponentially decaying function, which accounts for the slow decay of the instantaneous pulse. It does not however account for the time it take a synapse to process the incoming action potential, increasing instantaneously to the maximum value as soon as the spike arrives. The last example takes into account this synaptic processing delay, increasing smoothly to its peak value and then decaying exponentially back to zero. Note that Θ(*t*) represents the Heaviside function

For a single uncoupled neuron (*k* = 0) it is a simple matter to show that, when *v*_th_ →*∞* and *v*_reset_ →−*∞*, the frequency of oscillation is given by $2\sqrt {\eta _{i}}/C$. For further simplicity we shall work with these choices for the values of *v*_th_ and *v*_reset_. From now on we shall choose *η*_*i*_ to be random variables drawn from a Lorentzian distribution *L*(*η*) with

7 $$ L(\eta) = \frac{1}{\pi}\frac{\Delta}{(\eta-\eta_{0})^{2}+{\Delta}^{2}}, $$

where *η*_0_ is the centre of the distribution and Δ the width at half maximum. For simplicity we will consider the average frequency of oscillations $\omega _{0} =2\sqrt {\eta _{0}}/C$ as a single parameter, distributed with a width at half maximum ${\Delta }\omega =2\sqrt {\Delta }/C$. In the coupled network, and if the frequencies of the individual neurons are similar enough, then one may expect some degree of phase locking (ranging from synchrony to asynchrony), itself controlled in part by the time-to-peak, 1/*α*, of the synaptic filter.

**Fig. 5:**
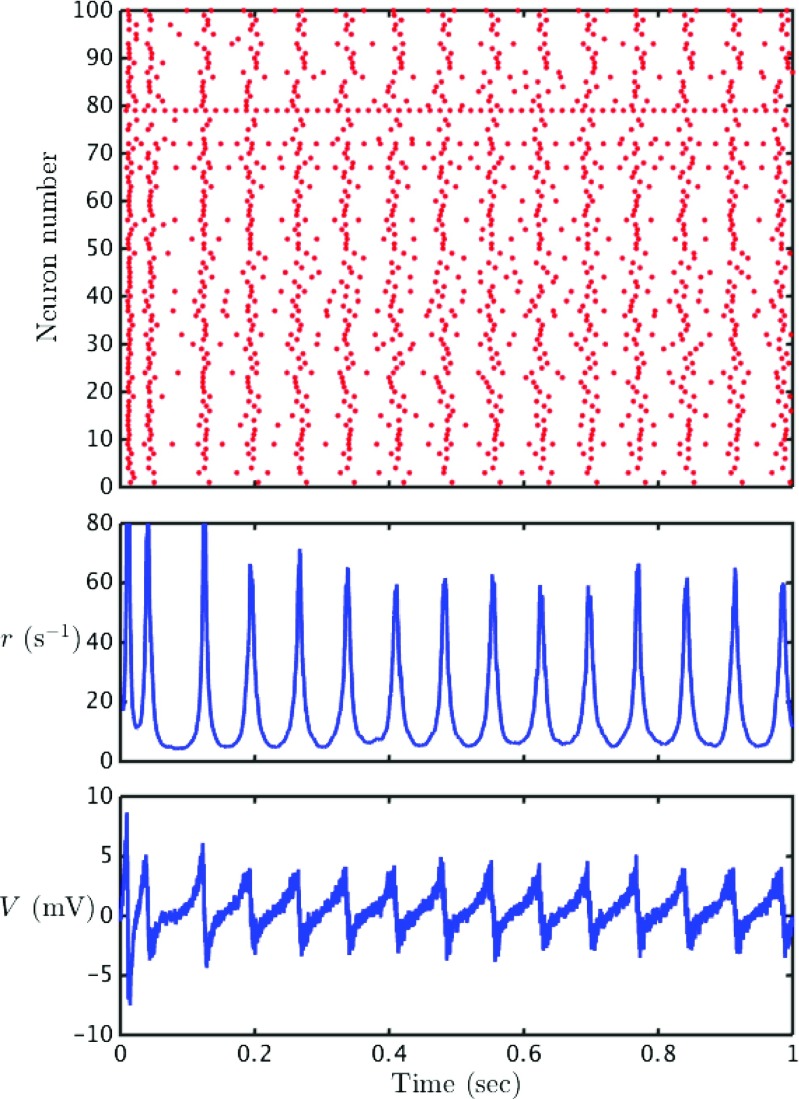
Quadratic integrate-and-fire dynamics: The *top plot* show a raster plot depicting the spike times for a sample of size 100 in a network of 500 QIF neurons. The *lower plots* show the mean field variables; the firing rate *r* and the average voltage *V*. Parameter values are chosen such that the system exhibits partial synchrony; *ω*_0_ = 0.269 Hz, Δ*ω* = 0.042 Hz, *v*_syn_ = −10 mV, *k* = 0.105, 1/*α* = 35 ms, *C* = 30 mF

As shown in Fig. 5, for a model with predominantly inhibitory connections, we see patterns of coherent spiking. The degree of coherence is mainly controlled by the degree of heterogeneity of the constant current drives *η*_*i*_. In this figure we also track the evolution of two macroscopic order parameters. These are respectively the average membrane potential, given by Eq. (6), and the instantaneous mean firing rate *r*:

8 $$ r(t) = \frac{1}{N} \sum\limits_{j=1}^{N} \sum\limits_{m \in \mathbb{Z}} \delta (t-{T_{j}^{m}}) . $$

For large *N* both the order parameters *V* and *r* show a smooth temporal variation. In the case of synchrony we would expect these mean field signals to show a periodic temporal variation, essentially following a trajectory reminiscent of a single QIF neuron receiving periodic drive, whilst for an asynchronously firing population these mean field signals would be constant in time (modulo finite size fluctuations). To quantify the degree of coherence (or phase-locking) within an oscillatory population it is convenient to use a Kuramoto order parameter. First though it is necessary to recast the model in terms of *phase* variables.

Given the well known link between the QIF neuron and the *θ*-neuron it is natural to introduce the phase variable *θ*_*i*_ ∈ [−*π*, *π*) according to *v*_*i*_ = tan(*θ*_*i*_/2) (so that $\cos \theta _{i} = (1-{v_{i}^{2}})/(1+{v_{i}^{2}})$ and $\sin \theta _{i} = 2v_{i}/(1+{v_{i}^{2}})$). In this case we arrive at the *θ*-neuron network

9 $$ C\frac{\mathrm{d} }{\mathrm{d} t}{\theta}_{i} = (1-\cos\theta_{i}) + (1+\cos\theta_{i})(\eta_{i} + gv_{\text{syn}}) - g\sin\theta_{i}, $$

10 $$ Q g = \frac{2}{C} \frac{k}{N} \sum\limits_{j=1}^{N} P(\theta_{j}). $$

Here *P*(*θ*) = *δ*(*θ* − *π*) and is periodically extended such that *P*(*θ*) = *P*(*θ* + 2*π*), and we have used the result that $\delta (t-{T_{j}^{m}}) = \delta (\theta _{j}(t)- \pi ) |\dot {\theta }_{j}({T_{j}^{m}})|$. The network defined by Eqs. (9) and (10) describes a set of *N* phase variables interacting via spike triggered currents every time that *θ*_*j*_ passes through *π*. We will only consider the case that *θ*_*j*_ increases through *π* (so that spikes are only generated on the upswing of the corresponding voltage variable). In the absence of synaptic coupling an isolated *θ*-neuron supports a pair of equilibria *θ*_±_, with *θ*_+_ < 0 and *θ*_−_ > 0 for *η*_*i*_ < 0, and no equilibria for *η*_*i*_ > 0. In the former case the equilibria at *θ*_+_ is stable and the one at *θ*_−_ unstable. In neurophysiological terms, the unstable fixed point at *θ*_−_ is a threshold for the neuron model. Any initial conditions with *θ* ∈ (*θ*_+_, *θ*_−_) will be attracted to the stable equilibrium, while initial data with *θ* > *θ*_−_ will make a large excursion around the circle before returning to the rest state. For *η*_*i*_ > 0 the *θ*-neuron oscillates with frequency $2\sqrt {\eta _{i}}/C$. When *η*_*i*_ = 0 the *θ*-neuron is poised at a saddle-node on an invariant circle (SNIC) bifurcation.

As well as naturally providing a phase variable the *θ*-neuron network is more straight forward to simulate as the model has continuous trajectories on an *N*-torus (and there is no need to handle the discontinuous reset conditions). The Kuramoto order parameter is then defined as

11 $$ Z(t) = \frac{1}{N}\sum\limits_{j=1}^{N} \mathrm{e}^{i\theta_{j}(t)} \equiv R(t) \mathrm{e}^{i{\Psi}(t)} . $$

**Fig. 6:**
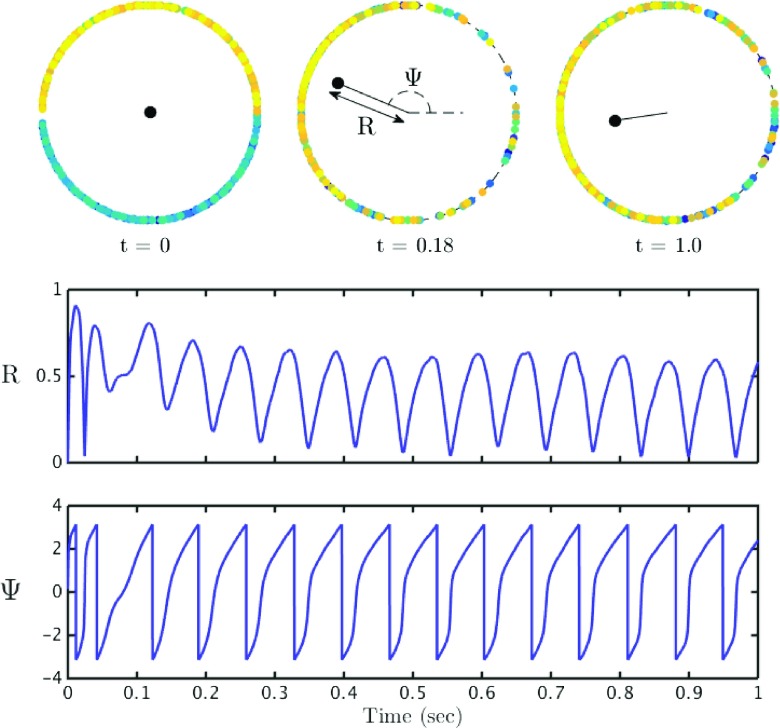
Theta neuron dynamics: The *top set of plots* show the phases of the individual neurons, represented by the coloured dots, at 3 different values of time *t*. The colour of each neuron has no intrinsic meaning, and is used merely to aid in distinguishing the dots. The phase of each neuron is the angular position of the coloured dot. The *black dot* in the centre represents the Kuramoto order parameter *Z* = *R* e^*i*Ψ^. In the *top left plot* the system is completely asynchronous and *Z* ≃ 0. The *top middle plot* demonstrates that the length from the centre of the disk to the *black dot* represents *R*, the population synchrony, and Ψ, the average phase, is represented by the angular position of the black dot. The *top right plot* shows the system at a later point in time. The *lower plots* show *R* and Ψ as a function of time. One observes that both the population synchrony and average phase continuously oscillate. Parameter values as in Fig. 5

Here *R* provides a measure of the degree of coherence within the network and Ψ is the average phase. If a population is perfectly synchronised then *R* = 1 and similarly if the system is perfectly asyncronous then *R* = 0. In Fig. 6 we show a sequence of snapshots of the Kuramoto order parameter for the dynamics shown in Fig. 5, as well as the time evolution of the degree of coherence.

**Fig. 7:**
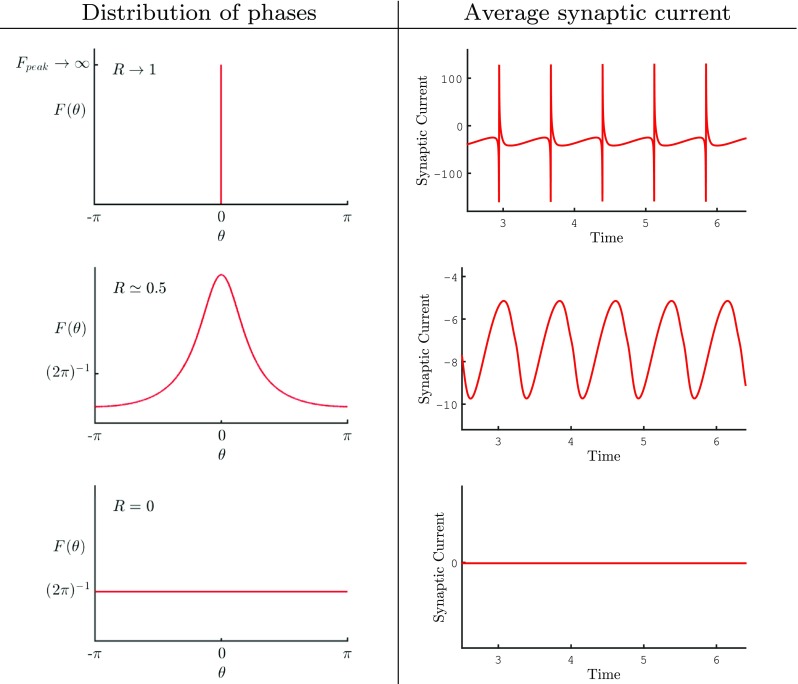
Distribution of phases: Figure illustrating the distribution of phases *F*(*θ*) in the large *N* limit and the average synaptic current for different values of the population coherence *R*. For simplicity we have fixed the choice of time so that Ψ(*t*) = 0. When the population is completely synchronous (*R* = 1) all of the neurons have the same phase and as a result all of the neurons fire together so that *F*(*θ*) = *δ*(*θ*) and the average synaptic current is very *spiky*. In the regime where *R* ≃ 0.5 the phases are more distributed. Although a dominant phase can be clearly identified (by the peak value) not all neurons have this phase. The OA ansatz gives the shape of the distribution in the form *F*(*θ*) = (2*π*)^−1^(1 −|*Z*|^2^)/(|e^*iθ*^ − *Z*|^2^). This spread in the phase distribution acts to smooth out the spikes in the average synaptic current to create a smooth oscillatory signal. When the population of neurons is completely asynchronous there is no dominant phase and every phase is equally probable so that *F*(*θ*) = 1/(2*π*). In this case all of the neurons fire at different times with their phases uniformly distributed to yield a constant synaptic current. Note that the peak in the distribution of phases move as the system evolves with time with a velocity $\dot {\Psi }$

Much as the order parameters (*r*, *V* ) vary smoothly with time (for large *N*) then so does the pair (*R*,Ψ). Indeed in the large *N* limit Luke et al. (2013), making use of the Ott-Antonsen (OA) ansatz (Ott and Antonsen 2008), have shown that a globally pulse-coupled network of *θ*-neurons has an exact mean field description. The OA ansatz allows the reduction of globally coupled phase oscillators in the infinite size limit to an explicit finite set of nonlinear ODEs. These describe the macroscopic evolution of the system, in terms of the Kuramoto order parameter for synchronisation, as long as the distribution of phases is at most single peaked. In essence the ansatz is well suited to describing systems which dynamically evolve between an incoherent asynchronous state and a partially synchronised state, which is often the case in systems with interactions that are prescribed by harmonic functions, such as found in Eq. (9). To illustrate the type of network evolution that can be generated with different values of synchrony, see Fig. 7. Here we show some plots of the phase distribution for different values of the network coherence as well as the average network current that would be produced.

Interestingly, Montbrió et al. have recently shown how to move between order parameters for the phase and voltage descriptions with the use of a conformal transformation (Montbrió et al. 2015). If we introduce the complex order parameter for the voltage description as

12 $$ W= \pi C r+iV , $$

then the following transformation allows one to switch perspectives and obtain the order parameter for the voltage description in terms of the Kuramoto order parameter as

13 $$ W= \frac{1-\overline{Z}}{1+\overline{Z}} , $$

where $\overline {Z}$ denotes the complex conjugate of *Z*. Importantly the OA ansatz can also be used to obtain a mean field model in the presence of non-pulsatile synaptic, extending the approaches in Luke et al. (2013) and Montbrió et al. (2015). In Appendix B we show that this yields the mean field model described by the fourth order ODE system:

14 $$\begin{array}{@{}rcl@{}} C \frac{\mathrm{d} }{\mathrm{d} t}Z &=& -i\frac{(Z-1)^{2}}{2}+\frac{(Z+1)^{2}}{2}\left[-{\Delta} + i \eta_{0} + i v_{\text{syn}} g \right] \\ &&- \frac{Z^{2}-1}{2} g , \end{array} $$

15 $$\begin{array}{@{}rcl@{}} Q g &=& k H(Z) . \end{array} $$

Here *H*(*Z*) is a global state dependent drive to the population given by

16 $$ H(Z) =\frac{1}{C\pi}\frac{1-\left|Z\right|^{2}}{1+Z+\overline{Z}+\left|Z\right|^{2}} , \qquad |Z| < 1 . $$

**Fig. 8:**
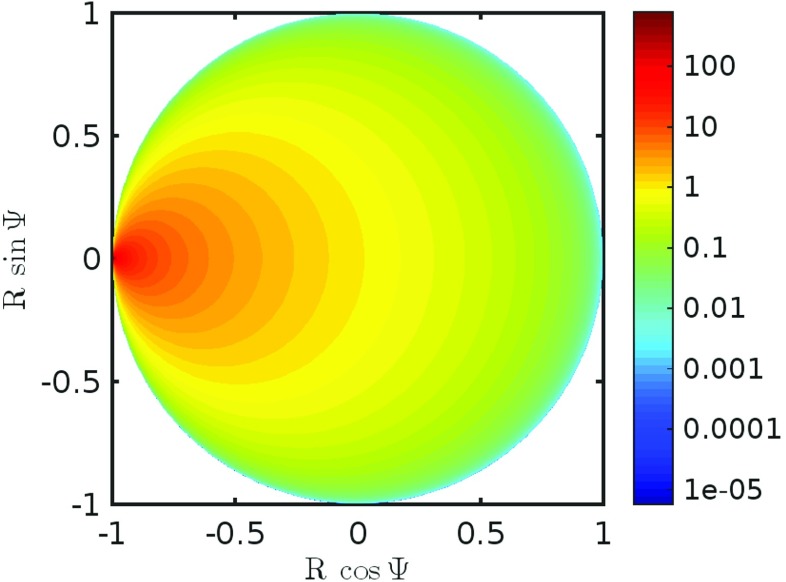
Plot of *H*: Density plot showing *H* as a function of the complex number *Z* = *R* e^*i*Ψ^. As *H* corresponds to the firing rate it takes a high value near *Z* = e^*iπ*^, this corresponds to highly synchronous behaviour where all of the phases of the neurons go through *π* simultaneously

A plot of this scalar function of a complex variable is shown in Fig. 8. It is illuminating to express *H* as function of *W* using (13) from which we find

17 $$ H(W) = \frac{1}{C\pi}\frac{W + \overline{W}}{2} = r . $$

Hence we may interpret *H*(*Z*) as the firing rate of the population that drives the global synaptic current. Figure 8 shows *H* as a function of *Z*. As expected *H* takes its highest value when *Z* ≃e^*iπ*^, corresponding to high synchrony where all of the neurons fire and reset at the same time.

**Fig. 9:**
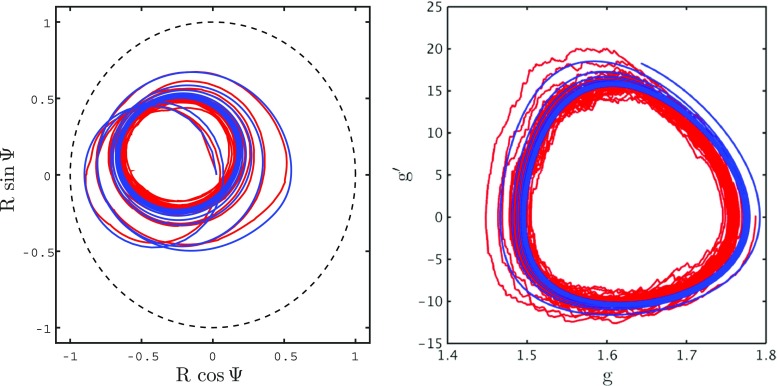
Validity of reduction: Comparison between the reduced mean field network (*blue*) and simulation a network of 500 *θ*-neurons (*red*). Phase plane for the Kuramoto order parameter *Z* = *R* e^*i*Ψ^ is shown on the *left* and the phase plane for the synaptic conductance *g* and its derivative $g^{\prime }$ is shown on the *right*. Parameter values as in Fig. 5

**Fig. 10:**
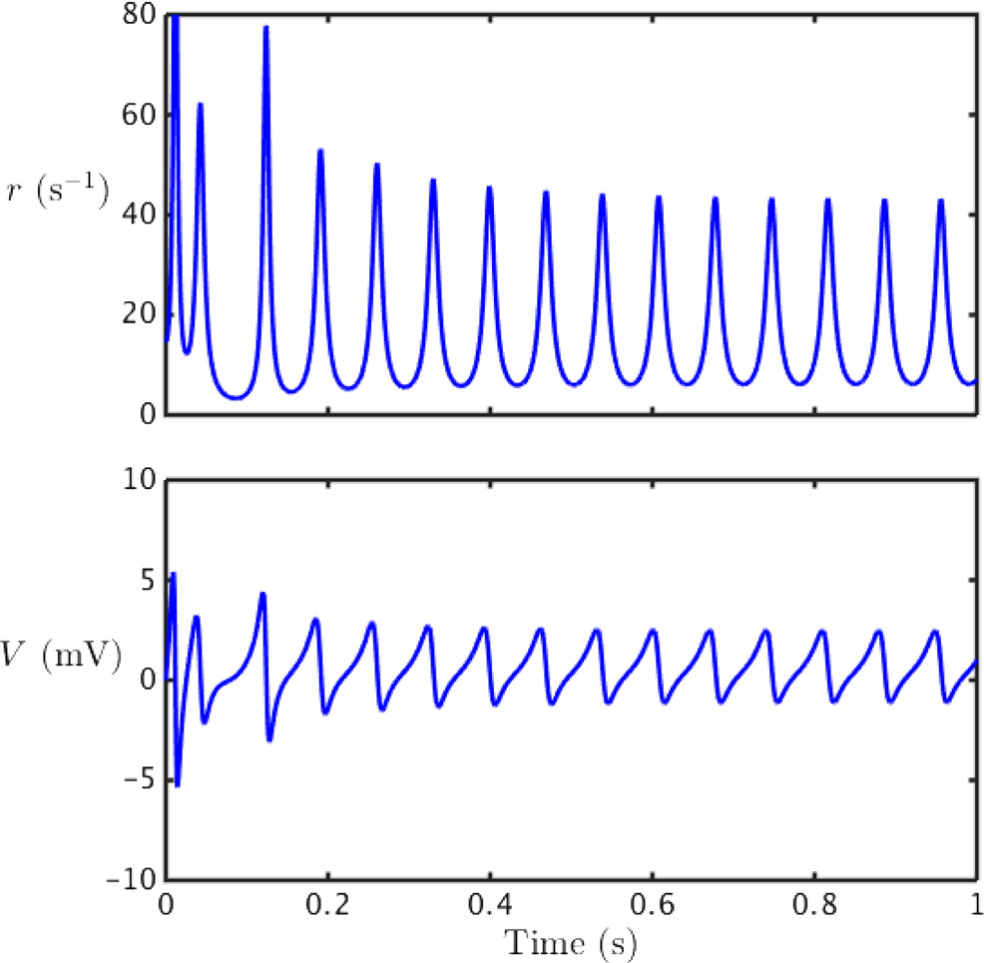
Mean field reduction for a QIF network: Time series for the mean field variable *W* = *π* *C* *r* + *i* *V*, where *r* is the population firing rate and *V* is the average voltage. Comparing these plots to the corresponding plots for a 500 neuron simulation in Fig. 5 it is clear to see that they agree well. The finite size fluctuation for *V* are quite apparent when comparing the results for the large scale simulation to those of the reduced mean field model. However, the overall behaviour is similar. Parameters as in Fig. 5

**Fig. 11:**
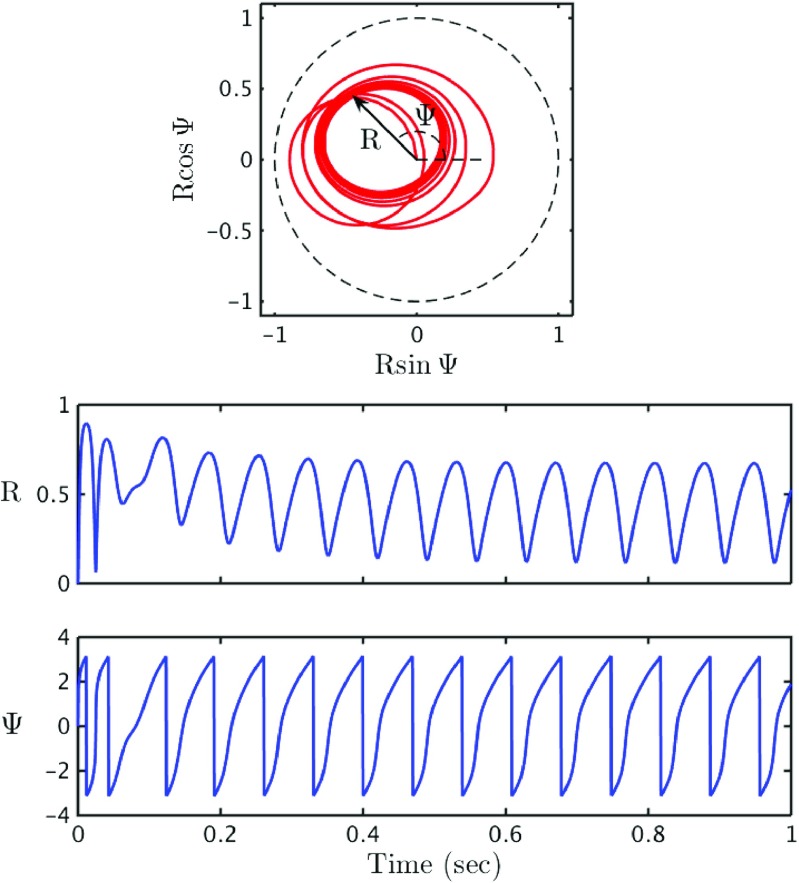
Mean field reduction for a *θ*-neuron network: Phase plane of the Kuramoto order parameter *Z*, showing *R*(*t*) and Ψ(*t*), as well as a time series for both *R* and Ψ. Once again the plots match very well with the corresponding plots for the simulation of 500 *θ*-neurons in Fig. 6. Interestingly even the initial behaviour is well matched. Parameter values as in Fig. 5

Figure 9 shows results for a simulation of 500 theta neurons (red) and a simulation of the reduced mean field model (blue). It is strikingly clear that the two simulations agree very well. If the size of the population in the large scale simulations is reduced then one can begin to see finite size fluctuations, as expected. The macroscopic order parameters (*r*, *V* ) in the reduced mean field model are plotted in Fig. 10. Unsurprisingly they behave similarly to the corresponding order parameters for the large scale simulations plotted in Fig. 5. Likewise, the mean field representation of (*R*,Ψ), plotted in Fig. 11, agree extremely well with those shown in Fig. 6. For a further discussion of the bifurcation structure of this model see Coombes and Byrne (2017).

## A mechanistic interpretation of movement induced changes in the beta rhythm

In Section 2 we demonstrated how an externally cued thumb movement caused a ∼ 0.5 s decrease in beta band power followed by a ∼ 2 − 4 s increase in beta band power, typifying MRBD and PMBR, respectively. The median nerve stimulation lasts ∼ 50 ms, however the evoked response lasts significantly longer. Upon examining the time-frequency spectrograms in Fig. 1 we observed an increase in low frequency activity at *t* = 0, which appears to last for ∼ 0.3 − 0.4 s, corresponding to the transduced median nerve stimulation and corresponding movement. We base the design of the external drive on this transduced signal.

We model the transduced signal as a temporally filtered drive *A* = *A*(*t*) that is received by every neuron in the model. In this case the dynamics of *Z* obey (14) under the replacement *η*_0_ → *η*_0_ + *A*, with *Q*_*D*_ *A*(*t*) = Ω(*t*), where *Q*_*D*_ is the differential operator obtained from *Q* in Eq. 5 under the replacement *α* → *α*_*D*_, and Ω(*t*) is a rectangular pulse, Ω(*t*) = πΘ(*t*)Θ(*τ* − *t*), where π can be interpreted as the strength of the drive. Note that the pulse is not applied until after transients have dropped off. As the evoked response in the experimental data last for ∼ 0.3 − 0.4 s we set *τ* = 0.4 s.

**Fig. 12:**
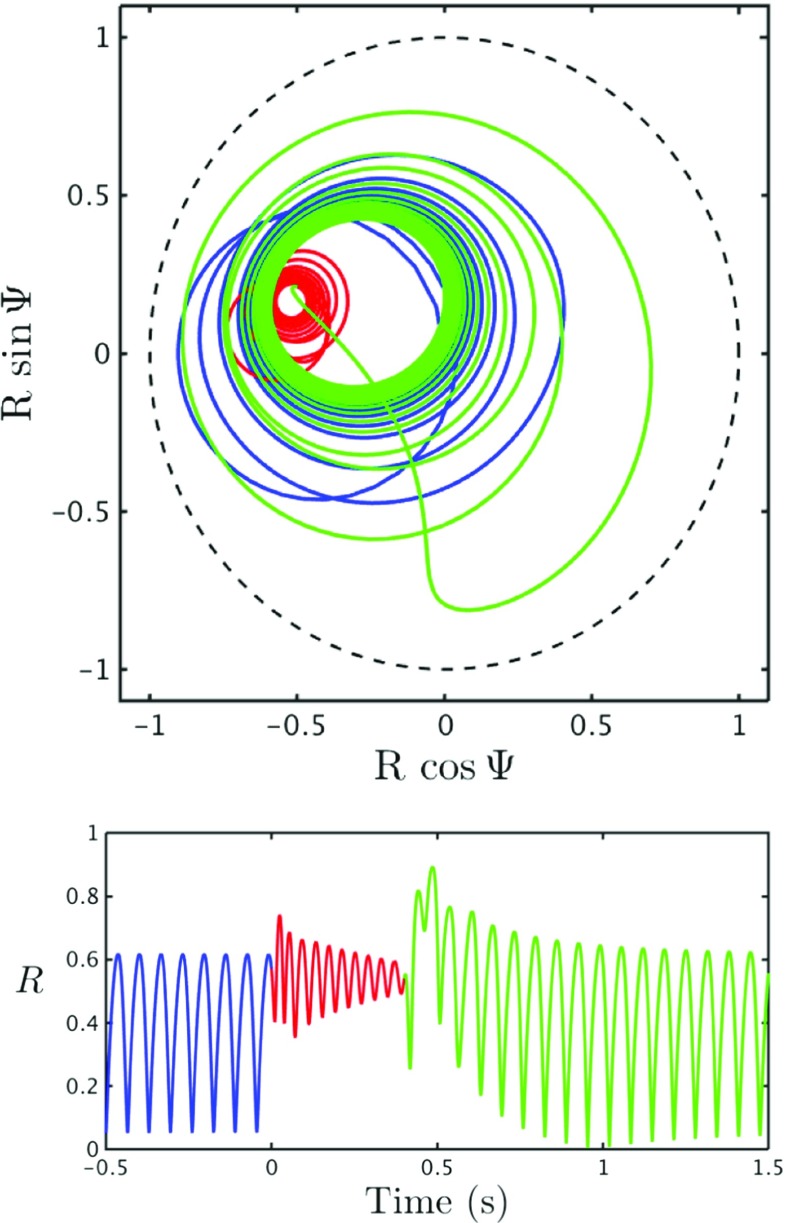
Response of *Z* to drive *Top*: Phase plane for *Z*, demonstrating the behaviour of the system in response to the drive *A*(*t*). The *blue curve* represents the system before the pulse arrives, as it settles to its non-perturbed dynamics (*t* < 0), the *red curve* demonstrates how the system behaves when the pulse is switched on (0 < *t* < *τ*) and the *green* how the system reacts once the drive is switched off (*t* > *τ* s). *Bottom*: Time series of *R* showing the change in the level of coherence, before, during and after the drive is switched on. The amplitude of the oscillations in *R* appear significantly reduced while the drive is switched on. Parameter values as in Fig. 5, apart from *ω*_0_ which was increased to 0.279 Hz, as this value gave a stronger PMBR, *τ* = 0.4, 1/*α*_*D*_ = 5.6 ms and π = 15 mA

Figure 12 shows the phase plane for the Kuramoto order parameter *Z* = *R* e^*i*Ψ^, as well as a time series for the within population synchrony *R*, in response to the drive described above. The colours correspond to the different time periods; before drive (blue), during drive (red), after drive (green). The system oscillates in partial synchrony with *R* oscillating between ∼ 0.05 − 0.6 in the absence of drive. Once the drive is switched on the amplitude of these oscillations decreases and hence the power is also reduced, corresponding to MRBD. Note that the frequency also increases during this period. After the drive is switched off the level of coherence is increased as *Z* is drawn towards the edge of the unit disk before spiralling back to the original limit cycle, corresponding to PMBR. Importantly the system does not rebound until *t* ≃ 0.5 s as seen in the real data. It should be noted that the stimulus corresponds to ∼ 80% of this time to rebound, however as the evoked response is present in ∼ 60 − 80% of the ∼ 0.5 s of MRBD in the real data we believe that this is a good fit.

**Fig. 13:**
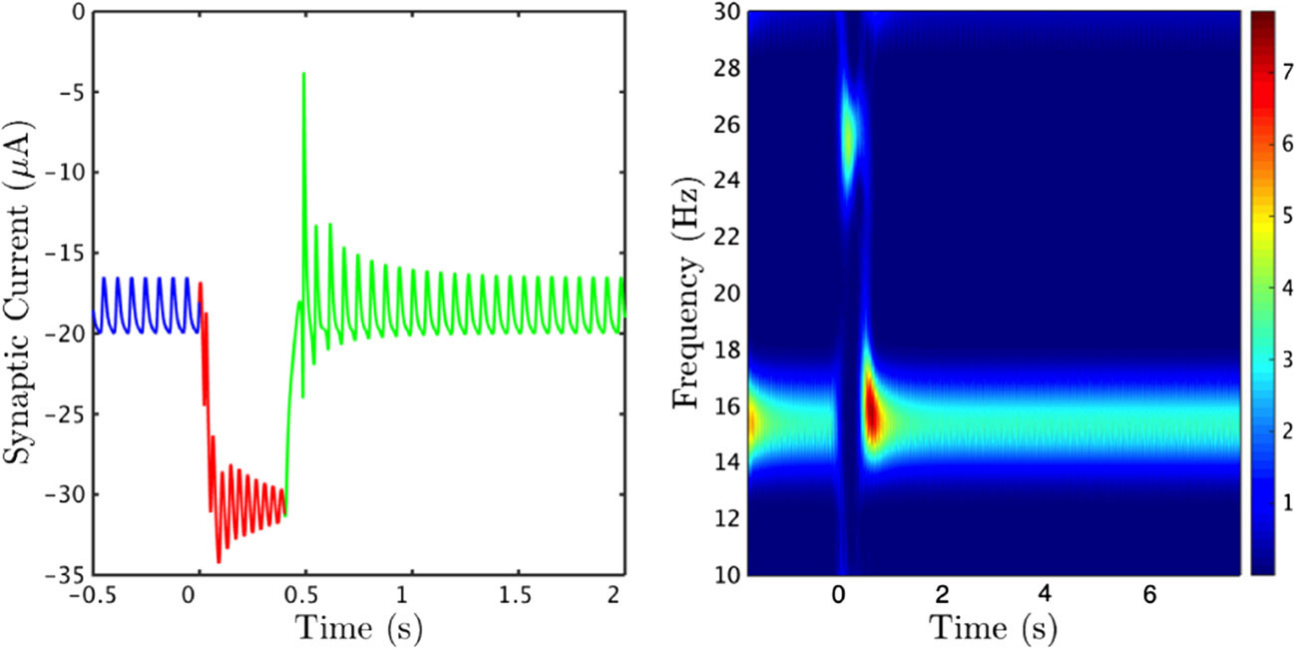
Response of the synaptic current to drive: Time series and spectrogram of the synaptic current showing the response of the system to the external drive Ω(*t*). The colours in the time series (*left*) correspond to the different time periods; before drive (*blue*), during drive (*red*), after drive (*green*). Both figures clearly demonstrate the rebound of the system, there is an increase in amplitude (and hence power) ∼ 0.5 s after the drive was initially switched on. Parameters as in Fig. 12

The corresponding response of the synaptic current is shown in Fig. 13. The time series (left) shows that when the drive is switched on the synaptic current is reduced, however the neurons are now also receiving a strong excitatory current in the form of the drive. There is a large increase in the amplitude of the oscillations of the synaptic current at ∼ 0.5 s, corresponding to PMBR, which can also be seen in the time-frequency spectrogram (right). The initial increase in amplitude is very large, however the percentage increase between 0.5 − 1.5 s is relatively small. The synaptic current appears to have fully settled back to its pre-drive behaviour by *t* ≃ 1.5 s, indicating a PMBR of roughly 1 s, which is not as long as the PMBR seen in our experimental data. An increase in power can be seen at around 26 Hz at *t* ≃ 0 s, corresponding to the increase in frequency during the drive on period. This high beta activity can be interpreted as the processing of the motor input.

Interestingly, we see a direct correlation between synchrony and the synaptic current. The time series in Fig. 12 (bottom) shows a peak in synchrony at *t* ≃ 0.5 s, just as the time series in Fig. 13 (left) shows a sharp increase in the amplitude of the synaptic current at *t* ≃ 0.5 s. This increase in amplitude can also be seen in the spectrogram (right). The strength of the drive π dictates the extent of the rebound. However it also prescribes the frequency of the oscillations amid the period when the drive is switched on. Therefore it is important to find the balance, where we have a prominent PMBR but also a physically realistic frequency during the interval when the stimulus is switched on. Although the increase in power at a higher frequency cannot be seen in our experimental data (Fig. 1), these time-frequency spectrograms were calculated for a small area of the motor cortex, it can be seen in the results obtained in Robson et al. (2015) (Fig. 2). It is widely believed that an increase in high beta and gamma activity is present in motor preparation and execution, in a more frontal region of the motor cortex.

The parameters were chosen such that the system oscillated at beta frequency and a significant MRBD and PMBR could be observed. The model is robust and can reproduce MRBD and PMBR for a wide region of parameter space.

## Discussion

We have presented a mechanistic model that exhibits both MRBD and PMBR. This low dimensional model is derived from a corresponding high dimensional spiking network model and maintains a faithful representation of synaptic currents. In the reduced model these currents are driven by a firing rate that is itself a function of the complex Kuramoto order parameter. This makes a significant departure from the usual phenomenological neural mass description of neuronal population dynamics for which the firing rate is usually only a function of synaptic activity or mean membrane potential. Importantly the transient response of the reduced model is sufficiently rich to capture the emergent time scales of both MRBD and PMBR, when it is stimulated whilst operating in the beta frequency range. Although the length of PMBR observed in the reduced model was shorter than that seen in the experimental data, it is still within the documented 1 − 10 s range. Given that model responses are linked to changes in within-population coherence, this gives further support to the notion that beta band amplitude changes, and in particular those in MRBD and PMBR, are in fact due to changes in synchrony. More generally the model parameters can be altered so that the population oscillates at other frequencies, and hence, used to explain other ERD/ERS phenomena in the brain.

One natural extension of the model is to small networks, with coupling through the mean-field variables of a set of local populations, to describe systems with a mixture of excitation and inhibition. This would lead to a richer set of structures within the phase space for the network and provide further mechanisms for controlling emergent time-scales (say as orbits can be made to approach saddle structures, leading to a slow down in dynamics), which may help lengthen the PMBR, see Coombes and Byrne (2017) for a discussion of the bifurcation structure for a two population model. An interesting study would be to couple two identical populations, corresponding to left and right motor cortex areas, and drive one of the populations to inspect the bilateral response as seen in experimental data (Robson et al. 2015; Liddle et al. 2016). It is also possible that noise may play a constructive role, and the OA ansatz for a mean-field reduction can also be performed in this case (Lai and Porter 2905). The introduction of noise may also result in a lengthening of the PMBR.

Perhaps of more interest in using these next generation neural models to replace existing neural mass descriptions, is the development of reductive techniques to handle other nonlinear integrate-and-fire models, such as those of Izhikevich type (Izhikevich 2003). However, this is a substantial mathematical challenge since the OA reduction technique that we have employed here will break down. However, it is possible to extend the work presented here to include gap junction coupling (Laing 2015). There is now little doubt that gap junctions play a substantial role in the generation of neural rhythms, both functional (Hormuzdi et al. 2004; Bennet and Zukin 2004) and pathological (Velazquez and Carlen 2000; Dudek 2002). It is also interesting to consider the spatially extended version of this model, we report on this elsewhere (Byrne et al. 2017).

Another possible extension would be to include a variety of different synaptic receptor. We have assumed that PMBR and MRBD are mediated by the same type of synaptic receptor. However, Hall et al. (2011) suggest that MRBD is a GABA-A mediated process, whilst PMBR appears to be generated by a non-GABA-A receptor mediated process. A further model that distinguishes between receptors, may offer important insights into motor processes, and can be readily accomplished within the framework that we have presented here.

## Appendix A: Experimental method

### A.1 Data collection

In order to demonstrate directly the robustness of beta band modulation in sensorimotor cortex, a somatosensory paradigm was used. Two subjects took part in the study, which was approved by the University of Nottingham Medical School Ethics Committee. The paradigm comprised electrical stimulation of the subject’s left median nerve, which was achieved by applying a series of 500 *μ* *s* duration current pulses to two gold electrodes placed on the subject’s wrist. The current was delivered using a Digitimer DS7A constant current stimulator, and the amplitude was increased slowly until a visible movement of the thumb was observed. Each experimental run comprised a total of 80 pulses delivered with an inter-stimulus-interval (ISI) of 10 s. A single experimental run lasted approximately 13 minutes. Each subject performed the study twice to assess robustness.

MEG data were captured using the third order synthetic gradiometer configuration of a 275-channel CTF whole-head MEG system (MISL, Port Coquitlam, Canada). The subject was positioned supine, with their head in the MEG helmet, whilst data were recorded at a 600 Hz sampling rate. Three localisation coils were attached to the head as fiducial markers (nasion, left preauricular and right preauricular) prior to the recording. Energising these coils at the start and end of data acquisition enabled localisation of the fiducial markers relative to the MEG sensor geometry as well as determination of total head movement. In order to co-register brain anatomy to the MEG sensor array, prior to the MEG recording each subject’s head shape was digitised relative to the fiducial markers using a 3D digitiser (Polhemus IsoTrack, Colchester, VT, USA). Volumetric anatomical MR images were acquired using a 3 T MR system (Phillips Achieva, Best, Netherlands) running an MPRAGE sequence (1 mm ^3^ resolution). Following data acquisition, the head surface was extracted from the anatomical MR image and coregistered (via surface matching) to the digitised head shape for each subject. This allowed complete coregistration of the MEG sensor array geometry to the brain anatomy, thus facilitating subsequent forward and inverse calculations.

### A.2 Data analysis

MEG data were inspected visually and any trials containing excessive interference removed. Data were then analysed using synthetic aperture magnetometry (SAM) (Vrba and Robinson 2001), a beamforming variant (van Drongelen et al. 1996; Gross et al. 2001; Hillebrand et al. 2005; Robinson and Vrba 1998; van Veen et al. 1997) that has been applied successfully in many studies to localise neural oscillatory amplitude changes. Data were first filtered to the beta (13–30 Hz) band. Following this the SAM beamformer was applied and oscillatory amplitude was contrasted in active and control time windows in order to delineate the spatial signatures of beta amplitude change. The forward model was based upon a multiple local sphere head model and the forward calculation by Sarvas (Huang et al. 1999; Sarvas 1987). Covariance was computed within the prescribed windows (Brookes et al. 2008). Pseudo-t-statistical images were generated.

## Appendix B: Mean field reduction

In the limit *N* → *∞* the state of the network at time *t* can be described by a continuous probability distribution function *ρ*(*η*, *θ*, *t*), which satisfies the continuity equation (arising from the conservation of oscillators):

18 $$ \frac{\partial \rho}{\partial t} +\frac{\partial \rho c}{\partial \theta} = 0, $$

where *c* is a given realisation of $\dot {\theta }$,

19 $$\begin{array}{@{}rcl@{}} c &=& \frac{1}{C} \left[(1-\cos\theta) + (1+\cos\theta)(\eta + g v_{\text{syn}})\right.\\ &&\quad~\left.- g\sin\theta \right]. \end{array} $$

The global drive to the network, given by the right hand side of Eq. (10), can be constructed as

20 $$ \lim_{N \rightarrow \infty} \frac{1}{N} \sum\limits_{j=1}^{N} P(\theta_{j}) = {\int}_{0}^{2 \pi} \mathrm{d} \theta {\int}_{-\infty}^{\infty} \mathrm{d} \rho(\eta,\theta,t) P(\theta) . $$

Hence,

21 $$\begin{array}{@{}rcl@{}} Q g &= &\frac{k}{\pi C} {\sum}_{m \in \mathbb{Z}} {\int}_{0}^{2 \pi} \mathrm{d} \theta {\int}_{-\infty}^{\infty} \mathrm{d} \rho(\eta,\theta,t) \mathrm{e}^{im (\theta-\pi)} , \end{array} $$

where we have used the result that $2 \pi P(\theta ) = {\sum }_{m \in \mathbb {Z}} \mathrm {e}^{im (\theta -\pi )}$. The formula for *c* above may be written conveniently in terms of e^±*i**θ*^ as

22 $$ c = \frac{1}{C} \left[ f \mathrm{e}^{i\theta} + h + \overline{f} \mathrm{e}^{-i\theta}\right], $$

where *f* = ((*η* − 1) + *v*_syn_ *g* + *i* *g*)/2 and *h* = (*η* + 1) + *v*_syn_ *g*, and $\overline {f}$ denotes the complex conjugate of *f*.

The OA ansatz assumes that *ρ*(*η*, *θ*, *t*) has the product structure *ρ*(*η*, *θ*, *t*) = *L*(*η*)*F*(*η*, *θ*, *t*) where *L*(*η*) is taken to be the Lorentzian given by Eq. (7). Since *F*(*η*, *θ*, *t*) should be 2*π* periodic in *θ* it can be written as a Fourier series:

23 $$ F(\eta,\theta,t) = \frac{1}{2 \pi} \left\{ 1 + \sum\limits_{n=1}^{\infty} F_{n} (\eta,t) \mathrm{e}^{i n \theta} + \text{cc} \right\}, $$

where cc denotes complex conjugate. The insight in Ott and Antonsen (2008) was to restrict the Fourier coefficients such that

24 $$ F_{n} (\eta,t) = \alpha(\eta,t)^{n}, $$

where |*α*(*η*, *t*)| ≤ 1 to avoid divergence of the series. There is also a further requirement that *α*(*η*, *t*) can be analytically continued from real *η* into the complex *η*-plane, and that this continuation has no singularities in the lower half *η*-plane, and that |*α*(*η*, *t*)| → 0 as Im *η* → −*∞*. If we now substitute (22) into the continuity Eq. (18), use the OA ansatz, and balance terms in e^*i**θ*^ we obtain an evolution equation for *α*(*η*, *t*) as

25 $$ \frac{\partial }{\partial t} \alpha + \frac{1}{C} \left( i \alpha^{2} f + i\alpha h + i \overline{f}\right) = 0 . $$

It is now convenient to remember the Kuramoto order parameter (11), which in the large *N* limit is given by

26 $$ Z(t) = {\int}_{0}^{2 \pi} \mathrm{d} \theta {\int}_{-\infty}^{\infty} \mathrm{d} \eta \rho(\eta,\theta,t) \mathrm{e}^{i \theta}. $$

We note that |*Z*| ≤ 1. Using the OA ansatz (and using the orthogonality properties of e^*i**θ*^, namely ${\int }_{0}^{2 \pi } \mathrm {e}^{i p \theta } \mathrm {e}^{i q \theta } \mathrm {d} \theta = 2 \pi \delta _{p+q,0}$) we then find that

27 $$ \overline{Z}(t) = {\int}_{-\infty}^{\infty} \mathrm{d} \eta L(\eta) \alpha(\eta,t) . $$

By noting that the Lorentzian (7) has simple poles at *η*_±_ = *η*_0_ ± *i*Δ the integral in Eq. (27) may be performed by choosing a large semi-circle contour in the lower half *η*-plane. This yields $\overline {Z}(t)=\alpha (\eta _{-},t)$, giving *Z*(*t*) = *α*(*η*_+_, *t*). Hence, the dynamics for *g* given by Eq. (21) can be written as

28 $$ Qg = k H(Z), $$

where

29 $$\begin{array}{@{}rcl@{}} H(Z) &=& \frac{1}{C\pi}\left\{1+\left[\sum\limits_{m=1}^{\infty} (-1)^{m} Z^{m} + \text{cc} \right]\right\} \end{array} $$

30 $$\begin{array}{@{}rcl@{}} &=& \frac{1}{C\pi}\frac{1-\left| Z\right|^{2}}{1+Z+\overline{Z}+\left|Z\right|^{2}}. \end{array} $$

with |*Z*| < 1. The dynamics for *Z* is obtained from Eq. (25) as

31 $$\begin{array}{@{}rcl@{}} C \frac{\mathrm{d} }{\mathrm{d} t} Z&=& -i\frac{(Z-1)^{2}}{2}+\frac{(Z+1)^{2}}{2}\left[-{\Delta} + i \eta_{0} + i v_{\text{syn}} g \right]\\ &&-\frac{Z^{2}-1}{2} g . \end{array} $$

## References

[CR1] Alegre M, Alvarez-Gerriko I, Valencia M, Iriarte J, Artieda J (2008). Oscillatory changes related to the forced termination of a movement. Clinical Neurophysiology: Official Journal of the International Federation of Clinical Neurophysiology.

[CR2] Ashwin P, Coombes S, Nicks R (2016). Mathematical frameworks for oscillatory network dynamics in neuroscience. Journal of Mathematical Neuroscience.

[CR3] Bennet MVL, Zukin RS (2004). Electrical coupling and neuronal synchronization in the mammalian brain. Neuron.

[CR4] Berger H (1929). Über das Elektroenzenkephalogram des Menschen. Archiv für Psychiatrie und Nervenkrankheiten.

[CR5] Brookes MJ, Vrba J, Robinson SE, Stevenson CM, Peters AM, Barnes GR, Hillebrand A, Morris PG (2008). Optimising experimental design for MEG beamformer imaging. NeuroImage.

[CR6] Brookes MJ, Wood JR, Stevenson CM, Zumer JM, White TP, Liddle PF, Morris PG (2011). Changes in brain network activity during working memory tasks: a magnetoencephalography study. NeuroImage.

[CR7] Brookes MJ, Liddle EB, Hale JR, Woolrich MW, Luckhoo H, Liddle PF, Morris PG (2012). Task induced modulation of neural oscillations in electrophysiological brain networks. NeuroImage.

[CR8] Byrne, A., Avitabile, D., & Coombes, S. (2017). A next generation neural field model: the evolution of synchrony within patterns and waves. *Physical Review E*. In prep. 10.1103/PhysRevE.99.012313 30780315

[CR62] Donner, T.H., & Siegel, M. (2011). A framework for local cortical oscillation patterns. *Trends in Cognitive Sciences*, *15*(5), 191–199. 10.1016/j.tics.2011.03.007 21481630

[CR9] Cassim F, Monaca CAC, Szurhaj W, Bourriez J-L, Defebvre L, Derambure P, Guieu J-D (2001). Does post-movement beta synchronization reflect an idling motor cortex?. Neuroreport.

[CR10] Cheyne DO (2013). MEG studies of sensorimotor rhythms: a review. Experimental Neurology.

[CR11] Coombes S (2010). Large-scale neural dynamics: simple and complex. NeuroImage.

[CR12] Coombes, S., & Byrne, A. (2017). *Lecture notes nonlinear dynamics in computational neuroscience: from physics and biology to ICT, chapter next generation neural mass models*. PoliTO Springer.

[CR13] Coombes, S., beim Graben, P., Potthast, R., & Wright, J. (Eds.) (2014). *Neural fields, theory and applications*. Berlin: Springer.

[CR14] Dudek FE (2002). Gap junctions and fast oscillations: a role in seizures and epileptogenesis?. Epilepsy Currents.

[CR15] Ermentrout GB, Kopell N (1986). Parabolic bursting in an excitable system coupled with a slow oscillation. SIAM Journal on Applied Mathematics.

[CR16] Gaetz W, Macdonald M, Cheyne D, Snead OC (2010). Neuromagnetic imaging of movement-related cortical oscillations in children and adults: age predicts post-movement beta rebound. NeuroImage.

[CR17] Gaetz W, Edgar JC, Wang DJ, Roberts TPL (2011). Relating MEG measured motor cortical oscillations to resting *γ*-aminobutyric acid (GABA) concentration. NeuroImage.

[CR18] Gross J, Kujala J, Hamalainen M, Timmermann L, Schnitzler A, Salmelin R (2001). Dynamic imaging of coherent sources Studying neural interactions in the human brain. Proceedings of the National Academy of Sciences of the United States of America.

[CR19] Hall SD, Stanford IM, Yamawaki N, McAllister CJ, Rönnqvist KC, Woodhall GL, Furlong PL (2011). The role of GABAergic modulation in motor function related neuronal network activity. NeuroImage.

[CR20] Hall SD, Prokic EJ, McAllister C, Ronnqvist KC, Williams AC, Yamawaki N, Witton C, Woodhall GL, Stanford IM (2014). Gaba-mediated changes in inter-hemispheric beta frequency activity in early-stage parkinson’s disease. Neuroscience.

[CR21] Hillebrand A, Singh KD, Holliday IE, Furlong PL, Barnes GR (2005). A new approach to neuroimaging with magnetoencephalography. Human Brain Mapping.

[CR22] Hipp JF, Hawellek DJ, Corbetta M, Siegel M, Engel AK (2012). Large-scale cortical correlation structure of spontaneous oscillatory activity. Nature Neuroscience.

[CR23] Hormuzdi SG, Filippov MA, Mitropoulou G, Monyer H, Bruzzone R (2004). Electrical synapses: a dynamic signaling system that shapes the activity of neuronal networks. Biochimica et Biophysica Acta.

[CR24] Huang MX, Mosher JC, Leahy RM (1999). A sensor-weighted overlapping-sphere head model and exhaustive head model comparison for MEG. Physics in Medicine and Biology.

[CR25] Izhikevich EM (2003). Simple model of spiking neurons. IEEE Transactions On Neural Networks.

[CR26] Jasper HH, Andrews HL (1936). Human brain rhythms. I. Recording techniques and preliminary results. Journal of General Physiology.

[CR27] Jasper HH, Andrews HL (1938). Brain potentials and voluntary muscle activity in man. Journal of Neurophysiology.

[CR28] Jasper HH, Penfield W (1949). Electrocorticograms in man: Effect of voluntary movement upon the electrical activity of the precentral gyrus. Archiv fü,r Psychiatrie und Nervenkrankheiten.

[CR29] Jensen O, Goel P, Kopell N, Pohja M, Hari R, Ermentrout B (2005). On the human sensorimotor-cortex beta rhythm: sources and modeling. NeuroImage.

[CR30] Jurkiewicz MT, Gaetz WC, Bostan AC, Cheyne D (2006). Post-movement beta rebound is generated in motor cortex: evidence from neuromagnetic recordings. NeuroImage.

[CR31] Kilavik BrE, Zaepffel M, Brovelli A, MacKay WA, Riehle A (2013). The ups and downs of β oscillations in sensorimotor cortex. Experimental Neurology.

[CR32] Klinshov VV, Teramae JN, Nekorkin VI, Fukai T (2014). Dense neuron clustering explains connectivity statistics in cortical microcircuits. PloS One.

[CR33] Lai YM, Porter MA (2905). Noise-induced synchronization, desynchronization, and clustering in globally coupled nonidentical oscillators. Physical Review E.

[CR34] Laing, C.R. (2015). Exact neural fields incorporating gap junctions. *SIAM Journal on Applied Dynamical Systems*, page to appear.

[CR35] Latham PE, Richmond BJ, Nelson PG, Nirenberg S (2000). Intrinsic dynamics in neuronal networks. I. Theory Journal of Neurophysiology.

[CR36] Liddle EB, Price D, Palaniyappan L, Brookes MJ, Robson SE, Hall EL, Morris PG, Liddle PF (2016). Abnormal salience signaling in schizophrenia: the role of integrative beta oscillations. Human Brain Mapping.

[CR37] Luke TB, Barreto E, So P (2013). Complete classification of the macroscopic behaviour of a heterogeneous network of theta neurons. Neural Computation.

[CR38] Mary A, Bourguignon M, Wens V, Op de Beeck M, Leproult R, De Tiège X, P. Peigneux. (2015). Aging reduces experience-induced sensorimotor plasticity. A magnetoencephalographic study. NeuroImage.

[CR39] Montbrió E, Pazó D, Roxin A (2015). Macroscopic description for networks of spiking neurons. Physica Review X.

[CR40] Muthukumaraswamy SD, Myers JFM, Wilson SJ, Nutt DJ, Hamandi K, Lingford-Hughes A, Singh KD (2013). Elevating endogenous GABA levels with GAT-1 blockade modulates evoked but not induced responses in human visual cortex. Neuropsychopharmacology: Official Publication of the American College of Neuropsychopharmacology.

[CR41] Ott E, Antonsen TM (2008). Low dimensional behavior of large systems of globally coupled oscillators. Chaos.

[CR42] Pazó D, Montbrió E (1009). Low-dimensional dynamics of populations of pulse-coupled oscillators. Physical Review X.

[CR43] Pfurtscheller G, Lopes da Silva FH (1999). Event-related EEG/MEG synchronization and desynchronization: basic principles. Clinical Neurophysiology.

[CR44] Pfurtscheller G, Solis-Escalante T (2009). Could the beta rebound in the EEG be suitable to realize a “brain switch”?. Clinical Neurophysiology.

[CR45] Pfurtscheller G, Stancák A, Neuper C (1996). Event-related synchronization (ERS) in the alpha band–an electrophysiological correlate of cortical idling: a review. International Journal of Psychophysiology.

[CR46] Pfurtscheller G, Neuper C, Brunner C, da Silva FL (2005). Beta rebound after different types of motor imagery in man. Neuroscience Letters.

[CR47] Pinotsis, D., Robinson, P., beim Graben, P., & Friston, K. (2014). Neural masses and fields: Modelling the dynamics of brain activity. *Frontiers in Computational Neuroscience 8*(149). 10.3389/fncom.2014.00149 PMC4237139 25477813

[CR48] Riehle, A., & Vaadia, E. (2004). *Motor cortex in voluntary movements: a distributed system for distributed functions*. CRC Press. ISBN 0203503589.

[CR49] Robinson, S.E., & Vrba, J. (1998). Functional neuroimaging by synthetic aperture magnetometry (SAM). In Yoshimoto, T., Kotani, M., Kuriki, S., Karibe, H., & Nakasato, N. (Eds.), *Recent advances in biomagnetism* (pp. 302–305): Tohoku University Press.

[CR50] Robson, S.E., Brookes, M.J., Hall, E.L., Palaniyappan, L., Kumar, J., Skelton, M., Christodoulou, N.G., Qureshi, A., Jan, F., Katshu, M.Z., Liddle, E.B., Liddle, P.F., & Morris, P.G. (2015). Abnormal visuomotor processing in schizophrenia. *NeuroImage: Clinical*. 10.1016/j.nicl.2015.08.005 PMC5107643 27872809

[CR51] Sarvas J (1987). Basic mathematical and electromagnetic concepts of the biomagnetic inverse problem. Physics in Medicine and Biology.

[CR52] Schnitzler A, Salenius S, Salmelin R, Jousmäki V, Hari R (1997). Involvement of primary motor cortex in motor imagery: a neuromagnetic study. NeuroImage.

[CR53] So P, Luke TB, Barretto E (2014). Networks of theta neurons with time-varying excitability Macroscopic chaos, multistability, and final-state uncertainty. Physica D.

[CR54] Solis-Escalante T, Müller-Putz GR, Pfurtscheller G, Neuper C (2012). Cue-induced beta rebound during withholding of overt and covert foot movement. Clinical Neurophysiology: Official Journal of the International Federation of Clinical Neurophysiology.

[CR55] Stancák A, Pfurtscheller G (1995). Desynchronization and recovery of beta rhythms during brisk and slow self-paced finger movements in man. Neuroscience Letters.

[CR56] Stevenson CM, Brookes MJ, Morris PG (2011). β-band correlates of the fMRI BOLD response. Human Brain Mapping.

[CR57] Timmermann L, Florin E (2012). Parkinson’s disease and pathological oscillatory activity: is the beta band the bad guy? - New lessons learned from low-frequency deep brain stimulation. Experimental Neurology.

[CR58] van Drongelen W, Yuchtman M, Van Veen BD, van Huffelen AC (1996). A spatial filtering technique to detect and localize multiple sources in the brain. Brain Topography.

[CR59] van Veen BD, van Drongelen W, Yuchtman M, Suzuki A (1997). Localization of brain electrical activity via linearly constrained minimum variance spatial filtering. IEEE Transactions on Bio-medical Engineering.

[CR60] Velazquez JLP, Carlen PL (2000). Gap junctions, synchrony and seizures. Trends in Neurosciences.

[CR61] Vrba J, Robinson SE (2001). Signal processing in magnetoencephalography. Methods (San Diego California).

